# Disrupted intrinsic functional brain topology in patients with major depressive disorder

**DOI:** 10.1038/s41380-021-01247-2

**Published:** 2021-08-12

**Authors:** Hong Yang, Xiao Chen, Zuo-Bing Chen, Le Li, Xue-Ying Li, Francisco Xavier Castellanos, Tong-Jian Bai, Qi-Jing Bo, Jun Cao, Zhi-Kai Chang, Guan-Mao Chen, Ning-Xuan Chen, Wei Chen, Chang Cheng, Yu-Qi Cheng, Xi-Long Cui, Jia Duan, Yiru Fang, Qi-Yong Gong, Wen-Bin Guo, Zheng-Hua Hou, Lan Hu, Li Kuang, Feng Li, Hui-Xian Li, Kai-Ming Li, Tao Li, Yan-Song Liu, Zhe-Ning Liu, Yi-Cheng Long, Bin Lu, Qing-Hua Luo, Hua-Qing Meng, Daihui Peng, Hai-Tang Qiu, Jiang Qiu, Yue-Di Shen, Yu-Shu Shi, Tian-Mei Si, Yan-Qing Tang, Chuan-Yue Wang, Fei Wang, Kai Wang, Li Wang, Xiang Wang, Ying Wang, Yu-Wei Wang, Xiao-Ping Wu, Xin-Ran Wu, Chun-Ming Xie, Guang-Rong Xie, Hai-Yan Xie, Peng Xie, Xiu-Feng Xu, Jian Yang, Jia-Shu Yao, Shu-Qiao Yao, Ying-Ying Yin, Yong-Gui Yuan, Yu-Feng Zang, Ai-Xia Zhang, Hong Zhang, Ke-Rang Zhang, Lei Zhang, Zhi-Jun Zhang, Jing-Ping Zhao, Rubai Zhou, Yi-Ting Zhou, Jun-Juan Zhu, Zhi-Chen Zhu, Chao-Jie Zou, Xi-Nian Zuo, Chao-Gan Yan

**Affiliations:** 1 Department of Radiology, The First Affiliated Hospital, College of Medicine, Zhejiang University, Hangzhou, Zhejiang China; 2 CAS Key Laboratory of Behavioral Science, Institute of Psychology, Chinese Academy of Sciences, Beijing, China; 3 Department of Psychology, University of Chinese Academy of Sciences, Beijing, China; 4 International Big-Data Center for Depression Research, Institute of Psychology, Chinese Academy of Sciences, Beijing, China; 5 Department of Rehabilitation Medicine, The First Affiliated Hospital, College of Medicine, Zhejiang University, Hangzhou, Zhejiang China; 6 Center for Cognitive Science of Language, Beijing Language and Culture University, Beijing, China; 7 Sino-Danish College, University of Chinese Academy of Sciences, Beijing, China; 8 Sino-Danish Center for Education and Research, Beijing, China; 9 Department of Child and Adolescent Psychiatry, NYU Grossman School of Medicine, New York, NY USA; 10 Nathan Kline Institute for Psychiatric Research, Orangeburg, NY USA; 11 Anhui Medical University, Hefei, Anhui China; 12 Beijing Anding Hospital, Capital Medical University, Beijing, China; 13 Department of Psychiatry, The First Affiliated Hospital of Chongqing Medical University, Chongqing, China; 14 The First Affiliated Hospital of Jinan University, Guangzhou, Guangdong China; 15 Department of Psychiatry, Sir Run Run Shaw Hospital, Zhejiang University School of Medicine, Hangzhou, Zhejiang, China; 16 The Second Xiangya Hospital of Central South University, Changsha, Hunan China; 17 First Affiliated Hospital of Kunming Medical University, Kunming, Yunnan China; 18 Department of Psychiatry, First Affiliated Hospital, China Medical University, Shenyang, Liaoning China; 19 Shanghai Mental Health Center, Shanghai Jiao Tong University School of Medicine, Shanghai, China; 20 Huaxi MR Research Center (HMRRC), Department of Radiology, West China Hospital of Sichuan University, Chengdu, Sichuan China; 21 Research Unit of Psychoradiology, Chinese Academy of Medical Sciences, Chengdu, Sichuan China; 22 Department of Psychosomatics and Psychiatry, Zhongda Hospital, School of Medicine, Southeast University, Nanjing, Jiangsu China; 23 Affiliated Mental Health Center & Hangzhou Seventh People’s Hospital, Zhejiang University School of Medicine, Hangzhou, Zhejiang, China; 24 Mental Health Center and Psychiatric Laboratory, West China Hospital of Sichuan University, Chengdu, Sichuan China; 25 Department of Clinical Psychology, Suzhou Psychiatric Hospital, The Affiliated Guangji Hospital of Soochow University, Suzhou, Jiangsu China; 26 The Institute of Mental Health, Second Xiangya Hospital of Central South University, Changsha, Hunan China; 27 Faculty of Psychology, Southwest University, Chongqing, China; 28 Department of Diagnostics, Affiliated Hospital, Hangzhou Normal University Medical School, Hangzhou, Zhejiang, China; 29 National Clinical Research Center for Mental Disorders (Peking University Sixth Hospital) & Key Laboratory of Mental Health, Ministry of Health (Peking University), Beijing, China; 30 Xi’an Central Hospital, Xi’an, Shaanxi China; 31 Department of Neurology, Affiliated ZhongDa Hospital of Southeast University, Nanjing, Jiangsu China; 32 Department of Psychiatry, The Fourth Affiliated Hospital, College of Medicine, Zhejiang University, Yiwu, Zhejiang China; 33 Institute of Neuroscience, Chongqing Medical University, Chongqing, China; 34 Chongqing Key Laboratory of Neurobiology, Chongqing, China; 35 Department of Neurology, The First Affiliated Hospital of Chongqing Medical University, Chongqing, China; 36 Center for Cognition and Brain Disorders, Institutes of Psychological Sciences, Hangzhou Normal University, Hangzhou, Zhejiang, China; 37 Zhejiang Key Laboratory for Research in Assessment of Cognitive Impairments, Hangzhou, Zhejiang, China; 38 First Hospital of Shanxi Medical University, Taiyuan, Shanxi China; 39 Laboratory of Psychological Health and Imaging, Shanghai Mental Health Center, Shanghai Jiao Tong University School of Medicine, Shanghai, China; 40 Department of Psychiatry, Shanghai Jiao Tong University School of Medicine, Shanghai, China; 41 State Key Laboratory of Cognitive Neuroscience and Learning & IDG/McGovern Institute for Brain Research, Beijing Normal University, Beijing, China; 42 Magnetic Resonance Imaging Research Center, Institute of Psychology, Chinese Academy of Sciences, Beijing, China

**Keywords:** Diagnostic markers, Neuroscience, Depression

## Abstract

Aberrant topological organization of whole-brain networks has been inconsistently reported in studies of patients with major depressive disorder (MDD), reflecting limited sample sizes. To address this issue, we utilized a big data sample of MDD patients from the REST-meta-MDD Project, including 821 MDD patients and 765 normal controls (NCs) from 16 sites. Using the Dosenbach 160 node atlas, we examined whole-brain functional networks and extracted topological features (e.g., global and local efficiency, nodal efficiency, and degree) using graph theory-based methods. Linear mixed-effect models were used for group comparisons to control for site variability; robustness of results was confirmed (e.g., multiple topological parameters, different node definitions, and several head motion control strategies were applied). We found decreased global and local efficiency in patients with MDD compared to NCs. At the nodal level, patients with MDD were characterized by decreased nodal degrees in the somatomotor network (SMN), dorsal attention network (DAN) and visual network (VN) and decreased nodal efficiency in the default mode network (DMN), SMN, DAN, and VN. These topological differences were mostly driven by recurrent MDD patients, rather than first-episode drug naive (FEDN) patients with MDD. In this highly powered multisite study, we observed disrupted topological architecture of functional brain networks in MDD, suggesting both locally and globally decreased efficiency in brain networks.

## Introduction

Major depressive disorder (MDD) is a widespread and debilitating psychiatric disorder that accounts for a significant share of illness-related disability around the world [1]. MDD is typified by chronic feelings of sadness, guilt, and worthlessness and an increased risk of suicide [2, 3]. However, the pathophysiological mechanisms underlying MDD remain elusive. A growing literature has conceptualized MDD as a disease reflecting abnormal functional integration of distributed brain regions that regulate both emotional and cognitive functions [4–6]. However, patterns of brain abnormalities in MDD have not been consistently reproducible due to limited sample sizes and flexibility of data analysis workflows in previous studies. Accordingly, we initiated the REST-meta-MDD consortium (http://rfmri.org/REST-meta-MDD), a coordinated multisite project that released the largest resting-state functional magnetic resonance imaging (R-fMRI) MDD dataset comprising over 1000 depressed patients and normal controls (NCs). Based on this highly powered sample, we reported decreased functional connectivity (FC) within the default mode network (DMN) in recurrent MDD [7], implicating abnormalities in the functional coupling of brain networks in the pathophysiology of MDD. Such robust functional alterations in MDD are crucial because they can provide a firm basis for potential biomarkers and the development of new treatment targets for MDD.

Brain functional networks exhibit topological properties that are intermediate between purely random and regular [8–11]. Specific topological features of brain networks, such as the combination of high local and global efficiency, are thought to support information processing and mental representations through segregated and integrated information processing [12]. Accordingly, MDD could reflect abnormalities in the topological features of functional brain networks (i.e., abnormal global and local efficiency) [13]. Previous studies have reported altered topology of the functional connectome in MDD, but the results have been inconsistent. Specifically, patients with MDD have been found to have increased global efficiency [14, 15] and local efficiency [16]. However, studies similar in scale and design have also reported decreased global and local efficiency [17–19] or no significant alterations [20–23]. Given these contradictory results, reproducible and reliable findings would be highly novel and provide solid foundations for the field. The lack of reproducibility may reflect the low statistical power of small sample sizes (N < 60 per group) and highly variable analytical pipelines (i.e., large number of “researcher degrees of freedom”) [24, 25]. A study’s capacity to detect true effects is limited by low power, while statistically significant findings in small sample size studies may not reflect true effects [26, 27]. A recent study found substantial variations in conclusions drawn by independent groups analyzing an identical dataset, showing the sizeable impact of analytical flexibility on scientific conclusions [28]. Another confounding factor may be the number of depressive episodes (first episode or recurrence) [29, 30]. Specifically, the topological structure of FC has been found to differ between first episode and patients with recurrent MDD [19].

Here, we aimed to use a highly powered multisite sample (REST-meta-MDD project, including >1000 MDD samples) [7] to reliably reveal the topological architecture of functional brain networks in MDD and investigate whether episode status contributes to topological abnormalities. To avoid excessive flexibility in data analysis, data were preprocessed at local sites using a standardized protocol, and the preprocessed time series were openly shared. To ensure the robustness of findings, we also tested various analysis strategies (e.g., different topological parameters, node definitions and head motion control strategies). We hypothesized that MDD would be characterized by abnormal topological features of functional networks (reduced global and local efficiency) and that such abnormalities would differ for patients with single and recurrent depressive episodes.

## Materials and methods

### Sample composition

We used R-fMRI data that come from the REST-meta-MDD consortium [7], which included 25 datasets of 2428 individuals (1300 MDD patients and 1128 NCs) from 17 hospitals. Among MDD patients, 562 were first episode patients with MDD and 282 were recurrent MDD patients (medication status and illness duration information were unavailable for the remaining patients). All participants underwent at least a T1-weighted structural scan and a R-fMRI scan. Table S1 shows the sample size and scanning parameters for each site. Following our previous study [7], we used incomplete information, spatial normalization with bad quality, poor coverage, large head motion, and sites with less than 10 individuals in either group as exclusion criteria. This yielded a sample of 821 MDD patients and 765 NCs from 16 datasets/sites (for details, see SI Methods). Only binary information regarding medication treatment was available. Among this sample, 527 patients provided information on medication usage, including 219 patients currently taking antidepressant medications. As expected, most patients were female (522 females vs. 299 males). With respect to subgroups, two research groups contributed data on 117 first-episode drug naïve (FEDN) patients as well as 72 recurrent MDD patients, five research groups contributed data on 227 FEDN patients and 388 NCs and 6 research groups contributed data on 189 patients with recurrent MDD and 423 NCs. All data have been deidentified and anonymized. Local Institutional Review Boards have approved all contributing studies. A written informed consent was signed by participants at each local institution.

### Data preprocessing

R-fMRI and structural MRI data were acquired and preprocessed at each site using the same DPARSF protocol [31] (SI Methods).

### Functional brain network construction

Nodes and edges between nodes make up a topological network. Brain nodes were defined using Dosenbach’s 160 atlas [32]. Brain edges were defined by FC between brain nodes. For each node, a sphere was created with a 5 mm radius, centered on the atlas coordinates. Then, the neural signal of each node was derived by averaging the preprocessed blood oxygen level-dependent (BOLD) signals of all voxels within the sphere. To derive the connectivity matrix of the brain, we computed Pearson correlation coefficients of BOLD signals between all pairs of nodes, which were then Fisher transformed to z values. For each subject, we calculated weighted topological parameters of the FC matrices over a wide range of network edge sparsities [33] (SI Methods).

### Network analysis

Global and nodal network metrics of the brain were calculated at each sparsity threshold with the Brain Connectivity Toolbox (downloaded from https://sites.google.com/site/bctnet/Home) [34]. Global network metrics included global efficiency (E_glob_) as well as local efficiency (E_loc_). Path length (L_p_) and clustering coefficient (C_p_) were used in validation analysis as they generally reflect the same information (for details, see SI Methods).

The area under the curve (AUC) across the sparsity range was calculated for each network measure. The AUC was selected for statistical analyses because of its superior sensitivity [33]. We grouped significant nodes according to a well-defined functional parcellation derived from 1000 healthy participants [35] and reported the brain networks to which they correspond. The Yeo atlas divided the human cortex into seven networks; of these, we used the following six: the somatomotor network (SMN), ventral attention network (VAN), visual network (VN), dorsal attention network (DAN), default mode network (DMN), and frontoparietal network (FPN). Note that the limbic network from Yeo et al. was not included in the present study because none of the 160 Dosenbach ROIs were located within this network. Instead, we defined subcortical ROIs as the “subcortical network,” one of the seven networks in our model.

### Statistical analysis

To control for potential systematic site-related confounding factors, we employed the linear mixed effect (LME) model to conduct statistical analyses: y ~ 1 + group + age + sex + education + head motion + (1 | site) + (group | site). In this LME model, the intercept and the group variable contained random effects specific to site and fixed effects independent of site. Other variables were considered covariates of no interest. The AUC was compared between patients with MDD and NCs for each global network measure and for each nodal network measure across 160 nodes. Multiple comparisons were corrected for with FDR correction. To further test relationships between network measures and symptom severity, the group variable in the LME model was replaced with HAMD scores.

In addition, subgroup analyses were conducted using the LME model. We compared the abovementioned network measures between FEDN MDD patients and NCs. Recurrent MDD patients and NCs as well as recurrent MDD patients and NCs were also compared.

### Validation analysis

We performed a number of validation analyses to test the robustness of our results. (1) Different topological parameters with equivalent meanings, i.e., C_p_ and L_p_ were evaluated. (2) We also verified results by additionally employing scrubbing (discarding time points which have framewise displacement > 0.2 mm) for head motion control, besides including the individual level Friston 24 model and the group level motion covariate in primary analyses. (3) Another functional atlas (i.e., Craddock’s functional clustering atlas [36]) was also used to construct functional brain networks. Further analyses on the effect of overall connectivity strength, medication treatment/illness duration and sex differences are provided in the SI Methods.

## Results

### Comparisons between all patients with MDD and NCs

#### Alteration of network topologies in patients with MDD

**Fig. 1:**
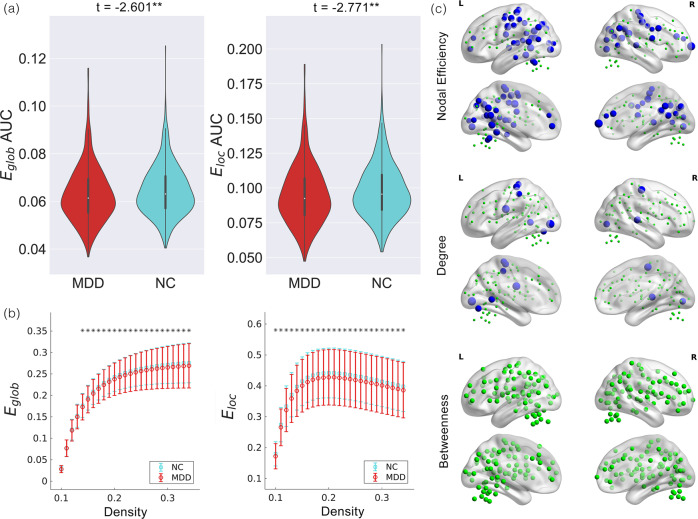
Group differences in network topological properties between major depressive disorder (MDD) patients and normal controls (NCs). **a** Violin plots illustrating the area under the curve (AUC) parameters of the global efficiency (E_glob_) and local efficiency (E_loc_) for MDD patients and NCs. Means and standard deviations are depicted. **b** E_glob_ and E_loc_ across a density range between 10% and 34%. Each point and error bar denote the mean and standard deviation at each density level, respectively. Asterisks indicate a significant difference at this density threshold. **c** Group differences in efficiency, degree and betweenness at the nodal level. Insignificant nodes are shown as green spheres, whereas blue (MDD < NC) and red (MDD > NC) spheres denote significant differences after FDR correction. The size of the significant nodes reflects the effect sizes of group differences. **: *p* < 0.01.

LME analyses revealed alterations in network properties in patients with MDD. Regarding network efficiency, E_glob_ (*t* = −2.601, *p* = 0.009) and E_loc_ (*t* = −2.771, *p* = 0.006) values were significantly decreased in patients with MDD compared to NCs (Fig. 1a and b).

#### MDD-related alterations in regional nodal features

Patients with MDD had a decreased nodal degree in the SMN (bilateral parietal lobe, left precentral gyrus and right temporal lobe), VN (bilateral occipital lobe) and DAN (left post parietal lobe) compared with NCs. We also found decreased nodal efficiency in the DMN (bilateral ventral medial prefrontal cortex (vmPFC), bilateral precuneus, bilateral posterior cingulate gyrus, bilateral angular gyrus, right ACC and intraparietal sulcus (IPS)), SMN (dorsal frontal cortex (dFC), right precentral gyrus, bilateral parietal and temporal lobe and posterior insula), DAN (left precentral gyrus, parietal lobe, inferior parietal lobe (IPL) as well as temporal parietal junction (TPJ)) and VN (bilateral occipital lobe) in patients compared with the nodal degree in these regions in NCs (Fig. 1c).

### Comparison between FEDN patients with MDD and NCs

#### Network topologies in FEDN patients and NCs

**Fig. 2:**
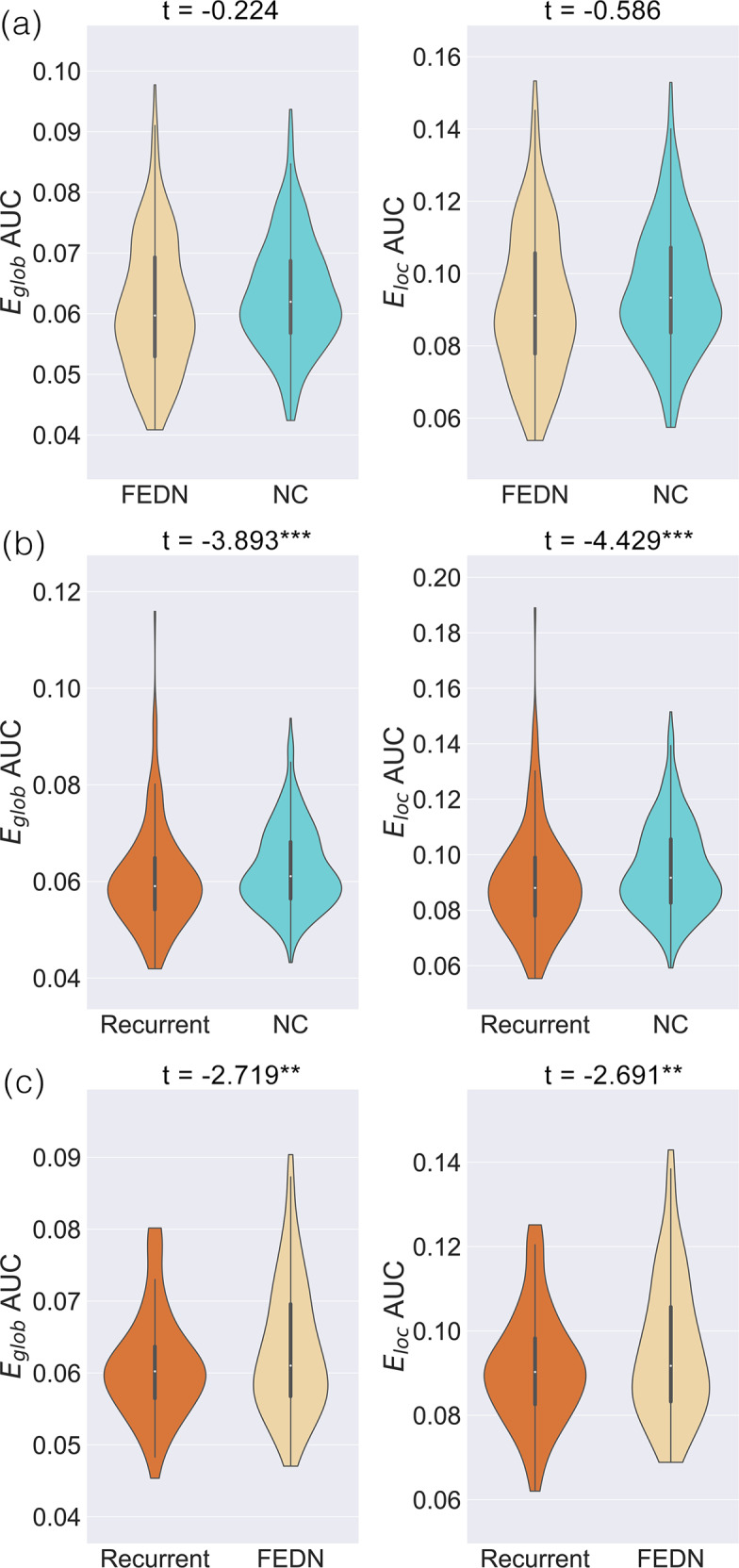
Subgroup differences in network topological properties (efficiency, E_glob_, and local efficiency, E_loc_). Distributions of areas under the curve (AUCs) are depicted. **a** First episode drug naïve (FEDN) patients with major depressive disorder (MDD) vs. normal controls (NCs). **b** Patients with recurrent MDD vs. NCs. **c** recurrent patients with MDD vs. FEDN patients. **: *p* < 0.01, ***: *p* < 0.001.

After sample selection, we compared the remaining 227 FEDN patients with 388 NCs from five research groups. No significant differences were revealed in network efficiency between FEDN patients and NCs (Fig. 2a and Figure S1a).

#### Alterations in regional nodal features in FEDN patients and NCs

**Fig. 3:**
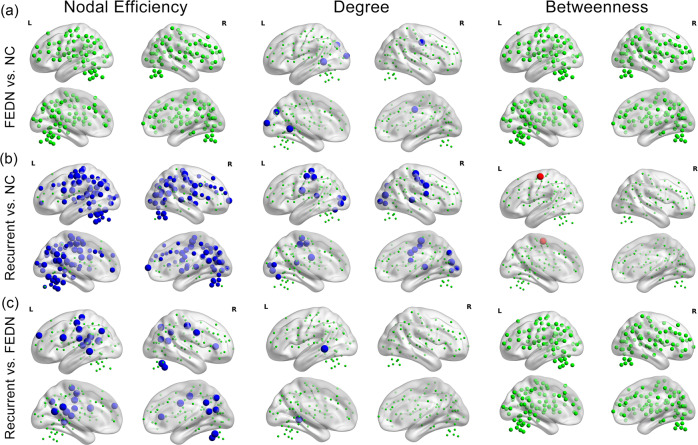
Subgroup differences in efficiency, degree and betweenness at the nodal level. Nonsignificant nodes are shown as green spheres. Blue (**a**: FEDN < NC; **b**: recurrent MDD < NC; **c**: recurrent MDD < FEDN) and red (**a**: FEDN > NC; **b**: recurrent MDD > NC; **c**: recurrent MDD > FEDN) spheres denote significant differences after FDR correction. The sizes of the significant nodes reflect the effect sizes of group differences. NC normal control, FEDN first-episode drug naïve.

Compared with NCs, nodal degree in FEDN MDD patients was decreased in the VN (left occipital lobe) and SMN (right precentral gyrus). No significant differences in nodal efficiency or betweenness were found between FEDN patients with MDD and NCs (Fig. 3).

### Comparisons between recurrent patients with MDD and NCs

#### Network topologies in recurrent patients with MDD and NCs

We found significantly decreased E_glob_ (*t* = −3.893, *p* < 0.001) and E_loc_ (*t* = −4.429, *p* < 0.001) values in recurrent patients compared with NCs (Figs. 2b and S1b).

#### Alterations in regional nodal features in recurrent patients and NCs

Compared with NCs, patients with recurrent depression showed decreased nodal degrees in a set of brain areas including the SMN (right frontal lobe, bilateral parietal lobe, precentral gyrus and temporal lobe) and VN (bilateral posterior occipital lobe); we also found decreased nodal efficiency in the DMN (bilateral vmPFC, right anterior cingulate cortex (ACC), bilateral precuneus and posterior cingulate cortex (PCC), bilateral angular gyrus, left inferior temporal lobe and left IPS), FPN (bilateral dorsal lateral prefrontal cortex (dlPFC), right anterior prefrontal cortex, bilateral IPL and right IPS), VAN (right anterior prefrontal cortex, medial and ventral frontal cortex, dorsal ACC and middle insula), SMN (bilateral precentral gyrus, bilateral middle insula, bilateral parietal lobe, bilateral temporal, and left post insula), and VN (bilateral occipital lobe) (Fig. 3).

### Comparisons between recurrent patients and FEDN patients

#### Network topologies in recurrent patients and FEDN patients

We found decreased E_glob_ (*t* = −2.719, *p* = 0.007) and E_loc_ (*t* = −2.691, *p* = 0.008) values in patients with recurrent MDD compared with the values found in FEDN patients (Figs. 2c and S1c).

#### Alterations in regional nodal features in recurrent patients and FEDN patients

Compared with FEDN patients, patients with recurrent MDD showed decreased nodal degree in the DMN (left inferior temporal lobe) and decreased nodal efficiency in the DMN (left anterior prefrontal cortex, bilateral PCC and precuneus, left angular gyrus and left inferior temporal lobe), DAN (left ventral prefrontal cortex and parietal lobe), SMN (right frontal lobe, left precentral gyrus, bilateral parietal lobe, left posterior insula and left temporal lobe), VAN (right ventral prefrontal cortex) and VN (right occipital lobe) (Fig. 3).

### Correlations between behavioral measures and network metrics

We also employed the LME model with HAMD score as the group variable to test correlations between symptom severity and network metrics. However, no results were significant after multiple comparison corrections.

### Validation analyses

**Fig. 4:**
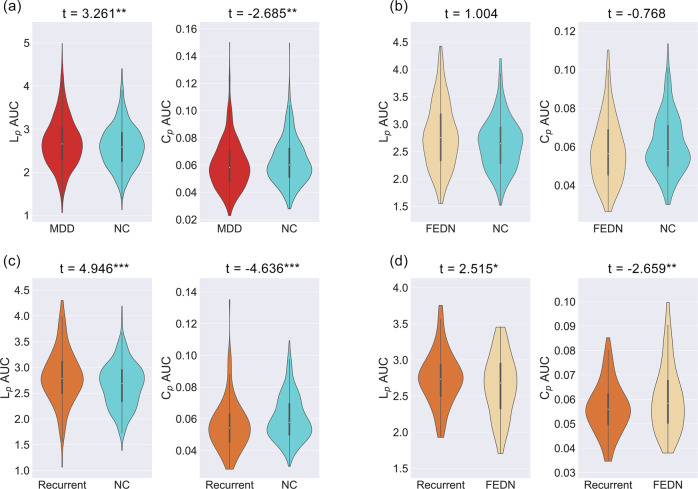
L_p_ and C_p_ differences between major depressive disorder (MDD) patients and normal controls (NCs) as well as subgroup contrasts. Distributions of areas under the curve (AUCs) are depicted. **a** MDD vs. NCs. **b** First-episode drug naïve (FEDN) patients with major depressive disorder (MDD) vs. normal controls (NCs). **c** Patients with recurrent MDD vs. NCs. **d** patients with recurrent MDD vs. FEDN patients. *: *p* < 0.05, **: *p* < 0.01, ***: *p* < 0.001.

Analysis of two confirmatory metrics (path length and clustering coefficient) confirmed our primary findings. Specifically, patients with MDD showed significantly higher path length values (*t* = 3.187, *p* = 0.001) and lower clustering coefficient values (*t* = −2.536, *p* = 0.011). We also found significantly enhanced path lengths (*t* = 4.969, *p* < 0.001) and decreased clustering coefficients (*t* = −4.631, *p* < 0.001) in recurrent patients with MDD compared to NCs, whereas FEDN subgroups did not differ significantly compared to NCs in these measures. Finally, recurrent patients with MDD also showed increased path lengths (*t* = 2.488, *p* = 0.014) and decreased clustering coefficients (*t* = −2.626, *p* = 0.009) compared to FEDN patients with MDD (see Fig. 4). Further validation analyses including scrubbing (Figs. S2–S3) and using an alternative brain parcellation (Figs. S4–S5) largely confirmed our main findings. We also found significant differences in overall FC between patients with MDD and NCs (Table S2) and some changes in results after controlling overall connectivity strength (Table S3). Marginally significant effects were revealed when comparing first episode patients on medication and FEDN patients, whereas no significant effects were found between patients with the longest and shortest illness durations (Table S4). In addition, patients with MDD showed no significantly abnormal topological properties compared to HCs after controlling medication usage (all ps > 0.05, Table S5). Although significant sex effects were revealed in the original model, there were no significant group-by-sex interaction effects when comparing all patients with MDD and HCs (Tables S6–S7). A more thorough description can be found in the SI Results.

## Discussion

Here, the topological architecture of functional brain networks was investigated in a large multisite sample of MDD patients and NCs through analysis with identical rigorous methods. We found altered topological network properties in patients with MDD, particularly decreased global and local efficiency compared with NCs. However, this result, which implicated an impairment of the normal integration of functional brain networks, was only significant in patients with recurrent MDD. Notably, at the nodal level, compared with NCs, we found decreased nodal degrees and nodal efficiency in several brain functional networks (the DMN, DAN, SMN, and VN) in MDD patients, which were especially prominent in patients with recurrent MDD.

Human brain topological organization generally features high local and global efficiency. High local efficiency benefits from densely clustered connections among topological neighbors, whereas high global efficiency reflects efficient information flow over the entire brain network [13]. This brain network architecture allows for efficient information separation and integration while using minimal wiring and energy. This kind of topological organization can be dramatically altered in neurological and psychiatric disorders [37]. Abnormal topological organization has been reported in MDD [16, 18, 38, 39], but the results have been strikingly inconsistent. Limited power from small sample size studies and variability in research methods may have contributed to the disparate results. Here, with standardized processing and a highly powered sample, we demonstrated that both global and local efficiency were significantly decreased in MDD and that this effect was mainly attributed to recurrent MDD patients. For decreasing the cost of information transport in brain networks, information coding is just as important as structural architecture. Neural information is processed online while passing within and between subnetworks. This online coding can be done by altering edge strengths (i.e., connectivity) within the network to dynamically integrate distributed nodes [40]. We hypothesize that in patients with recurrent MDD, lower information coding capacity is linked to lower local and global efficiency, reducing the effectiveness of processes that the local interactions to be organized to cope with a variety of environmental demands and to ensure robustness, adaptability, as well as resilience to distress.

Patients with MDD showed decreased nodal degrees and efficiency in regions belonging to the DMN, DAN, SMN, and VN, which are involved in cognitive executive processes, emotional processing, and basic sensory/motor functions. Evidence from both task-based [41] and resting-state studies has implicated abnormal visual processing in patients with MDD [42]. One recent study also reported that disruptions in the visual network were linked to clinical symptoms in MDD [43]. For the SMN, decreased regional homogeneity in depressed patients was found in a meta-analysis [30], which could explain psychomotor retardation, a key clinical symptom of MDD. Furthermore, the distributed abnormalities in the VN and SMN have been reported to be associated with MDD in a resting-state dynamic FC study [44, 45]. In summary, these findings could be interpreted as the neurological basis of psychomotor retardation’s extensive influence on attentional functions [46].

MDD studies have highlighted the DMN, which has been associated with rumination [47, 48], self-referential processing [49] and emotional appraisal [50, 51]. In the current study, we found decreased nodal efficiency in patients with MDD. However, further analysis revealed this decrease was only evident in recurrent patients with MDD and was not found in FEDN patients, in line with our previous study [7]. Greater social dysfunction among patients with MDD has been linked to diminished DMN connectivity [51], which pinpoints alterations in DMN connections as potentially germane to social dysfunction in MDD [52]. In our study, decreased nodal efficiency within the DMN suggests a weakened coordinating role, presumably in response to accumulating pathology from recurrent episodes of MDD.

Supplementary subgroup analysis found no alterations in topological properties in the FEDN subgroup. In addition, the group contrast between all MDD patients and HCs was no longer significant after including medication usage as a covariate. These results suggest that the abnormalities in recurrent MDD patients may be largely due to medication effects. Antidepressant medications have been found to effectively alter the resting-state FCs of patients with MDD [53, 54]. Our results indicated that antidepressant medications may also exert effects on topological properties. Moreover, we note that more information including medication type, dose, duration, and even adverse effects (and reasons for changing or ceasing medications more generally) are needed to better disentangle medication effects on the pathophysiology underlying MDD. Sex differences in MDD must be considered when interpreting the results of this study. Previous studies have identified depression-related sex differences in brain networks [55, 56]. We found that men showed higher global and local efficiency than women but no group-by-sex interactions were observed. Future large-scale studies focusing on the sex differences regarding brain networks in MDD should be conducted.

The strengths of the present study include the highly powered sample size, standardized preprocessing pipeline, and robustness across various analysis workflows. In addition, beyond having already shared the data through the R-fMRI Maps Project, we also openly shared the analysis code for the current brain topological study, thus allowing readers to validate or reuse our codes (https://github.com/Chaogan-Yan/PaperScripts/tree/master/Yang_2021_MolecularPsychiatry).

Several limitations must be noted. First, all participants were Chinese, so applicability to other ethnic/racial and cultural contexts must be confirmed. Second, our study was retrospective and cross-sectional, so we could not dissociate disease chronicity from medication effects. Prospective longitudinal studies including studies of remission and recurrence throughout the lives of MDD patients are needed. Third, substantial evidence has indicated that patients with MDD exhibit subtle but widespread deficits in fractional anisotropy of white matter [57–59]. Therefore, the topological organization of anatomical connectivity may be another potential biomarker for MDD. A highly powered anatomical connectivity sample has been created (i.e., The ENIGMA-MDD DTI Working Group), and further studies are needed to delineate reliable topological properties of anatomical connections in MDD patients. Fourth, since it has been well established that socioeconomic status is associated with the pathophysiology of MDD [60], further studies with more comprehensive socioeconomic data are needed. Finally, we note that MDD is a highly heterogeneous disorder [61]. Though the present study is based on traditional diagnostic criteria, future studies adopting the Research Domain Criteria (RDoC) framework [62] could inform the brain patterns that are associated with core dimensions of functioning (e.g., abnormal hedonic processing, threat sensitivity, etc.).

With a highly powered multisite sample, we showed that recurrent MDD is related to abnormalities in the topological architecture of functional brain networks, suggesting that the dampened global and local efficiency caused by this disruption may have a role in the pathology of MDD and may become more pronounced with recurrent episodes of MDD.

## Supplementary information

Supplementary Information
