# Supplementary material for: Disrupted intrinsic functional brain topology in patients with major depressive disorder
Source: Mol Psychiatry. 2021 Aug 12;26(12):7363–71. doi: 10.1038/s41380-021-01247-2 (PMC8873016; doi:10.1038/s41380-021-01247-2)
Supplement: Supplementary file 1 — Supplementary Information [file 41380_2021_1247_MOESM1_ESM.docx]

**Supplementary Information**

**SI Methods**

**Sample composition**

We selected subjects for topological and group statistical analyses using the following criteria: 1) Site 25 was excluded because its sample mainly consisted of patients with late onset MDD (most aged > 60 years) and remitted patients, resulting in 1,211 MDD patients and 1,064 NCs; 2) subjects lacking information on sex, age and education were excluded, resulting in 1,150 MDD patients and 971 NCs; 3) subjects with poor imaging data or bad spatial normalization were excluded by visual inspection, resulting in 1,042 MDD patients and 884 NCs; 4) subjects aged less than 18 years or more than 65 years were excluded, resulting in 989 MDD patients and 860 NCs; 5) subjects with bad coverage (<90% of the group mask) or excessive head motion (average framewise displacement (FD) > 0.2 mm) were excluded, resulting in 943 MDD patients and 846 NCs; 6) subjects with spatial correlation < 0.6 (a threshold defined by mean - 2SD) between each participant’s regional homogeneity (ReHo) map and the group mean ReHo map were excluded to further remove subjects with distortions that were not detected by visual inspection, resulting in 900 MDD patients and 815 NCs; and 7) sites with fewer than 10 patients with MDD or NCs in either group we removed, resulting in 848 MDD patients and 794 NCs from 17 sites. Furthermore, site 4 (including 18 patients with MDD and 23 NCs) was excluded because it was a duplicate of site 14 (detected during the preparation of REST-meta-MDD data for open sharing), resulting in 830 MDD patients and 771 NCs from 16 sites. Finally, we excluded subjects who did not have full functional coverage of all 160 ROIs. On average, each site contributed 51.3 ± 55.6 MDD patients (ranging from 16 to 246) and 47.8 ± 50.5 NCs (ranging from 14 to 225). Of note, the selection procedure of subgroups ensured that each MDD patient could be paired with an NC from the same site, given our primary interest in the contrast of patients with MDD vs. NCs. Substantial differences across different sites have been shown to dramatically dampen the generalizability of one site’s findings to a different site^1, 2^.

**Functional brain network construction**

The sparsity threshold, S, ranged from 0.10 to 0.34, with an interval of 0.01. Correlation thresholds ranged from 0.42 to 0.47 (mean ± SD = 0.44 ± 0.01) at S = 0.10 and from 0.25 to 0.28 (mean ± SD = 0.26 ± 0.01) at S = 0.34 across all subjects. We adopted a sparsity threshold to alleviate the effects of thresholding on the final topological metrics^3^ and because this strategy has been shown to be less influenced by subjects’ head motion ^4^. Density thresholds were chosen to discern prominent topological properties in brain networks (high global and local efficiency)^4^.

**Data preprocessing**

The initial 10 functional images were discarded. After that, slice acquisition timing discrepancies and head motion were corrected in functional images. Linear trends, Friston 24 head motion parameters^5^, the white matter signal and the cerebrospinal fluid signal were regressed out from the functional signal as nuisance covariates. Of note, we did not perform global signal regression due to the attendant controversy^6^. The Diffeomorphic Anatomical Registration Through Exponentiated Lie algebra (DARTEL) tool^7^ was used to normalize functional images to MNI space. We finally applied bandpass temporal filtering (0.01 – 0.1 Hz) to the normalized functional images.

**Network metrics**

Global efficiency (Eq. (1)) was defined as how efficiently the whole network exchanges information computed as:

 (1)

where E_i_ was node i’s weighted efficiency and d_ij_^w^ was the shortest weighted path length between nodes i and j. d_ij_^w^ was defined as:

 (2)

where a_uv_ was the connection status; a_ij_ = 1 as a link (i, j) existed (i.e., node i and j are neighbors); otherwise a_ij_ = 0 (a_ii_ = 0 for all i). w_uv_ stood for the connection weights between nodes u and v. f corresponded to a map from weight to length (i.e., an inverse mapping). g_i↔︎j_^w^ denoted the shortest weighted path between node i and j. N was the set of all nodes, and the number of nodes in the given network was denoted by the letter n. The superscript “w” indicated that this metric was calculated with weighted networks. Unless otherwise stated, all metrics used in the present study were weighted, so the superscript “w” was omitted in other sections.

Local efficiency was defined as the mean efficiency of the local subnetworks^8^ and computed as:

 (3)

where E_loc,i_^w^ was the weighted local efficiency of node i. The connection weights between nodes i and j were denoted by w_ij_, and d_jh_^w^(N_i_) was the weighted length of the shortest path between j and h, composed exclusively of the neighbors of i.

We also used two topological parameters with similar meanings as validation analyses, i.e., path length (Lp) and clustering coefficient (Cp). Characteristic path length (Eq. (4)) was equivalent to the inverse of E_glob_ and can be defined as:

 (4)

where L_i_^w^ was the mean weighted distance between node i and all the rest nodes. d_ij_^w^ was the shortest weighted length of path between i and j.

The clustering coefficient (Eq. (5)) was defined as the fraction of the neighbors of a node that are also neighbors of each other^9^ and was chosen to reflect local efficiency because it generally conveys similar information as E_loc_; it can be described as:

 (5)

where C_i_^w^ was the node i’s weighted clustering coefficient (C_i_^w^ = 0 if k_i_ < 2), k_i_ was the node i's degree, and t_i_^w^ was the geometric mean of triangles in the vicinity of i, which was computed as:

 (6)

where w_ij_ stood for the connection weights between i and j.

We calculated metrics of nodal centrality for each of the 160 nodes, including degree, nodal efficiency, and betweenness^10^. Degree (Eq. (7)) was the sum of links’ weights connected to a node. Nodal efficiency (Eq. (8)) was defined as inversely proportional to the mean shortest weighted distance between node i and all the rest nodes j of the network. Betweenness (Eq. (9)) was defined as the fraction of all shortest paths in the graph that pass through a certain node. The equations were computed as:

 (7)


 (8)


 (9)

where ρ_hj_ stood for the number of shortest paths between node h and j, and ρ_hj_ (i) was the number of shortest paths between node h and j that passed through i.

**Testing of Assumptions**

To test whether our data met the assumptions of the tests, we calculated the skewness and kurtosis of the distribution of the residuals from the original LME model of each topological metrics. All metrics’ skewness values were within range ± 2 and their kurtosis values were within range ± 7, which met the assumption of our statistical tests^11^. Furthermore, we also checked the similarity of variance in our contrasts with MATLAB’s command vartest2. For example, variances of C_p_ showed no significant differences (F = 1.112, p = 0.135).

**Visualization**

Nodal level abnormalities were rendered on the brain with BrainNet Viewer^12^.

**Validation analysis**

It could be argued that the overall connectivity strength may be a confounding factor in the investigation of topological properties. Therefore, we first compared the whole-brain mean functional connectivity (FC) between all MDD patients and NCs as well as each subgroup contrast. Then, we included the whole-brain averaged FC as an additional covariate in the original LME model to validate the robustness of our primary results. Furthermore, to examine whether abnormalities of recurrent patients were due to effects of medication treatment or illness duration, we compared first episode MDD patients on medication (113 MDD patients from site 20) with FEDN MDD patients (95 MDD patients from site 20). We also compared the tercile of patients with longest illness duration (≥ 24 months, 110 MDD patients from four sites) and those with shortest illness duration (≤ 6 months, 182 MDD patients from the same four sites). Furthermore, we also compared topological metrics of all MDD patients and HCs and included medication usage as an additional covariate. To test potential sex differences, we first checked the sex effect in the original LME model; then, we included a linear group-by-sex interaction as an additional covariate and tested its effects.

**SI Results**

To verify the robustness of our findings, we also included scrubbing in our preprocessing pipeline. As Figures S2 and S3 showed, the overall results were largely confirmed. We found reduced E_glob_ (t = -2.721, p = 0.007) and E_loc_ (t = -2.925, p = 0.004) values in MDD patients compared to NCs. We found a significant effect in recurrent MDD patients (E_glob_: t = -3.948, p < 0.001; E_loc_: t = -4.467, p < 0.001) but not in FEDN patients with MDD (E_glob_: t = -0.282, p = 0.778; E_loc_: t = -0.617, p = 0.537). Recurrent patients with MDD showed reduced E_glob_ values (t = -2.761, p = 0.006) and E_loc_ values (t = -2.736, p = 0.007) as compared to those of FEDN patients with MDD. Furthermore, the increased L_p_ values (t = 3.261, p = 0.001) and decreased C_p_ values (t = -2.685, p = 0.007) also differed significantly in patients with MDD compared to those of NCs. Again, this effect was significant only in recurrent patients with MDD (L_p_: t = 4.946, p < 0.001; C_p_: t = -4.636, p < 0.001) but not in FEDN patients with MDD (L_p_: t = 1.004, p = 0.316; C_p_: t = -0.768, p = 0.443). Recurrent patients with MDD also showed significantly increased L_p_ values (t = 2.515, p = 0.013) and decreased C_p_ values (t = 2.659, p = 0.009) compared to the FEDN MDD patients (see Figure S3).

Using an alternative brain parcellation that divided the brain into 200 regions (Craddock 200 atlas) largely confirmed our main findings (Figure S4 and S5). We found significantly decreased E_glob_ (t = -2.177, p = 0.030) and E_loc_ (t = -2.279, p = 0.023) values in patients with MDD compared to NCs. Patients with MDD also showed enhanced L_p_ (t = 2.552, p = 0.011) and reduced C_p_ (t = -2.099, p = 0.036) compared to NCs. No significant difference was found in FEDN patients with MDD compared to NCs, whereas recurrent patients with MDD had decreased E_glob_ values (t = -3.625, p < 0.001), E_loc_ values (t = -3.949, p < 0.001) and C_p_ values (t = -4.019, p < 0.001) values as well as increased L_p_ values (t = 3.896, p < 0.001) compared to NCs. Compared with FEDN patients with MDD, recurrent patients with MDD showed decreased E_glob_ (t = 2.594, p = 0.010), E_loc_ (t = 2.553, p = 0.011) and C_p_ (t = -2.441, p = 0.016) as well as enhanced L_p_ (t = 2.449, p = 0.015) values.

As overall functional connectivity could confound topological properties, we further tested the impacts of differences in general FC strength among different groups. As Table S2 shows, significant differences in general FC strength were revealed in the recurrent-NC contrast (t = -2.924, p = 0.004) and FEDN-recurrent MDD contrast (t = 2.495, p = 0.014). We then tested group differences in topological metrics after controlling overall connectivity strength. The results of the MDD-NC contrast and the recurrent MDD-NC contrast were generally in line with the original findings. Intriguingly, we found significant differences in Eloc, Lp and Cp between FEDN patients and NCs. In addition, differences between the FEDN and recurrent MDD groups were no longer significant after controlling for overall connectivity strength (Table S3). When comparing first-episode patients on medication and FEDN patients, we found marginally significant differences in E_glob_ (t = -1.769, p = 0.078), E_loc_ (t = -1.871, p = 0.063), L_p_ (t = 1.823, p = 0.070) and C_p_ (t = -2.018, p = 0.045) values. No significant differences were revealed between patients with the longest and shortest illness durations (Table S4). In addition, patients with MDD showed no significantly abnormal topological properties compared to HCs after controlling for medication usage (E_glob_ (t = -1.270, p = 0.204), E_loc_ (t = -1.481, p = 0.139), L_p_ (t = 1.734, p = 0.083) and C_p_ (t = -1.435, p = 0.152), see Table S5). Regarding sex differences, we found significant sex effects in E_glob_ (t = -3.591, p < 0.001), E_loc_ (t = -3.562, p < 0.001), L_p_ (t = 3.187, p < 0.001) and C_p_ (t = -3.303, p < 0.001) values using the original LME model (Table S6). However, no significant group-by-sex interaction effects were revealed in E_glob_ (t = 0.385, p = 0.700), E_loc_ (t = 0.221, p = 0.825), L_p_ (t = 0.206, p = 0.837) or C_p_ (t = -0.342, p = 0.732) values.

**SI Discussion**

We failed to find any abnormalities of regional nodal characteristics regarding the FPN, a critical large-scale brain network involved in the pathophysiology of MDD^13^. In a meta-analysis, Kaiser and her colleagues^14^ reported that the FC between the FPN and DMN was enhanced, whereas FC between the FPN and DAN was decreased in MDD patients, which implies that the functional coupling of the FPN may differ among brain networks in MDD. Therefore, it is possible that the topological properties of the FPN were influenced by both hyperconnections and hypoconnections between the FPN and different brain networks, which may have cancelled each other out.

Another notable finding is that no alterations in the degree of DMN nodes were found in patients with MDD. The degree corresponds to the number of existing links connected to a given node ^10^. Therefore, this metric not only reveals functional coupling within the DMN but also shows FCs between the DMN and other brain networks. In our prior study, we failed to find significantly abnormal FCs between the DMN and other brain networks^15^. Therefore, this finding implies that reduced coupling in patients with MDD mainly occurred within the DMN, whereas the coupling between the DMN and other brain networks was largely unaffected.

The deficits in brain topological organization in patients with MDD we showed were generally moderate, which is in line with other previous multisite, large-sample studies^16^. Schmaal et al.^17^ found moderately thinner cortical gray matter in MDD patients than in HCs (Cohen’s d: − 0.10 to − 0.14) after analyzing 2,148 MDD patients and 7957 HCs. Similarly, van Velzen et al.^18^ examined white matter alterations in a sample of 1,305 MDD patients and 1,602 HCs and found subtle but widespread white matter abnormalities (Cohen’s d: 0.12 to 0.26). Therefore, it is possible that the high power of our large sample made it possible to detect subtle alterations in MDD that had been neglected in previous small sample size studies.

A body of literature has investigated the topological features of functional brain networks in FEDN patients, but inconsistent results have been reported. Two studies^19, 20^ reported increased global efficiency, while one group^21^ found reduced small-worldness in FEDN patients compared to NCs. This inconsistency may indicate that abnormal topological architectures of functional brain networks in FEDN patients are complex and subtle, which is in line with the present study. Intriguingly, we found significant alterations in topological properties in FEDN patients compared to NCs only when overall FC strength was regressed out as a covariate. These findings indicate that the absence of differences between the FEDN and NC groups may be partly attributed to overall connectivity strength. Nevertheless, we speculate that simple group differences in overall connectivity strength that do not matter because overall connectivity strength did not differ significantly between the FEDN and NC group. Future studies could delineate the association between overall connectivity strength and topological properties in FEDN patients.

**References**

1. Friedman L, Stern H, Brown GG, Mathalon DH, Turner J, Glover GH *et al.* Test-retest and between-site reliability in a multicenter fMRI study. *Hum Brain Mapp* 2008; **29**(8)**:** 958-972.

2. Chen X, Lu B, Yan CG. Reproducibility of R-fMRI metrics on the impact of different strategies for multiple comparison correction and sample sizes. *Hum Brain Mapp* 2018; **39**(1)**:** 300-318.

3. Fornito A, Zalesky A, Breakspear M. Graph analysis of the human connectome: promise, progress, and pitfalls. *Neuroimage* 2013; **80:** 426-444.

4. Yan CG, Craddock RC, He Y, Milham MP. Addressing head motion dependencies for small-world topologies in functional connectomics. *Frontiers in human neuroscience* 2013; **7:** 910.

5. Friston KJ, Williams S, Howard R, Frackowiak RSJ, Turner R. Movement-Related effects in fMRI time-series. *Magnetic resonance in medicine* 1996; **35**(3)**:** 346-355.

6. Murphy K, Fox MD. Towards a Consensus Regarding Global Signal Regression for Resting State Functional Connectivity MRI. *Neuroimage* 2016.

7. Ashburner J. A fast diffeomorphic image registration algorithm. *NeuroImage* 2007; **38**(1)**:** 95-113.

8. Latora V, Marchiori M. Efficient Behavior of Small-World Networks. *Physical Review Letters* 2001; **87**(19)**:** 198701.

9. Watts DJ, Strogatz SH. Collective dynamics of ‘small-world’networks. *nature* 1998; **393**(6684)**:** 440.

10. Rubinov M, Sporns O. Complex network measures of brain connectivity: uses and interpretations. *Neuroimage* 2010; **52**(3)**:** 1059-1069.

11. George D, Mallery P. *IBM SPSS statistics 26 step by step: A simple guide and reference*. Routledge2019.

12. Xia M, Wang J, He Y. BrainNet Viewer: a network visualization tool for human brain connectomics. *PLoS One* 2013; **8**(7)**:** e68910.

13. Menon V. Large-scale brain networks and psychopathology: a unifying triple network model. *Trends in Cognitive Sciences* 2011; **15**(10)**:** 483-506.

14. Kaiser RH, Andrews-Hanna JR, Wager TD, Pizzagalli DA. Large-scale network dysfunction in major depressive disorder: A meta-analysis of resting-state functional connectivity. *JAMA psychiatry* 2015; **72**(6)**:** 603-611.

15. Yan CG, Chen X, Li L, Castellanos FX, Bai TJ, Bo QJ *et al.* Reduced default mode network functional connectivity in patients with recurrent major depressive disorder. *Proc Natl Acad Sci U S A* 2019.

16. Schmaal L, Pozzi E, T CH, van Velzen LS, Veer IM, Opel N *et al.* ENIGMA MDD: seven years of global neuroimaging studies of major depression through worldwide data sharing. *Transl Psychiatry* 2020; **10**(1)**:** 172.

17. Schmaal L, Hibar DP, Samann PG, Hall GB, Baune BT, Jahanshad N *et al.* Cortical abnormalities in adults and adolescents with major depression based on brain scans from 20 cohorts worldwide in the ENIGMA Major Depressive Disorder Working Group. *Mol Psychiatry* 2016.

18. van Velzen LS, Kelly S, Isaev D, Aleman A, Aftanas LI, Bauer J *et al.* White matter disturbances in major depressive disorder: a coordinated analysis across 20 international cohorts in the ENIGMA MDD working group. *Mol Psychiatry* 2019.

19. Guo H, Cheng C, Cao X, Xiang J, Chen J, Zhang K. Resting-state functional connectivity abnormalities in first-onset unmedicated depression. *Neural Regen Res* 2014; **9**(2)**:** 153-163.

20. Zhang J, Wang J, Wu Q, Kuang W, Huang X, He Y *et al.* Disrupted brain connectivity networks in drug-naive, first-episode major depressive disorder. *Biol Psychiatry* 2011; **70**(4)**:** 334-342.

21. Jin C, Gao C, Chen C, Ma S, Netra R, Wang Y *et al.* A preliminary study of the dysregulation of the resting networks in first-episode medication-naive adolescent depression. *Neurosci Lett* 2011; **503**(2)**:** 105-109.

Supplementary Table S1. Samples of selected sites in the present study, modified from Supplementary Table S3 of Yan et al.^15^

| Serial number* | Research groups | NCs (n) | MDD patients (n) | Scanner | Coil | TR (ms) | TE (ms) | Flip angle (degree) | Thickness/gap | Slice number | Time points | Voxel size | FOV |
| --- | --- | --- | --- | --- | --- | --- | --- | --- | --- | --- | --- | --- | --- |
| 1 | National Clinical Research Center for Mental Disorders (Peking University Sixth Hospital) & Key Laboratory of Mental Health, Ministry of Health (Peking University) | 74 | 74 | Siemens Tim Trio 3T | 32 channel | 2000 | 30 | 90 | 4.0 mm/0.8 mm | 30 | 210 | 3.28 × 3.28 × 4.80 | 210 × 210 |
| 2 | Department of Clinical Psychology, Suzhou Psychiatric Hospital, The Affiliated Guangji Hospital of Soochow University | 30 | 30 | Philips Achieva 3T | 8 channel | 2000 | 30 | 90 | 4.0 mm/0 mm | 37 | 200 | 1.67 × 1.67 × 4.00 | 240 × 240 |
| 7 | Sir Run Run Shaw Hospital, Zhejiang University School of Medicine | 38 | 49 | GE discovery MR750 | 8 channel | 2000 | 30 | 90 | 3.2 mm/0 mm | 37 | 184 | 2.29 × 2.29 × 3.20 | 220 × 220 |
| 8 | Department of Psychiatry, First Affiliated Hospital, China Medical University | 75 | 75 | GE Signa 3T | 8 channel | 2000 | 30 | 90 | 3.0 mm/0 mm | 35 | 200 | 3.75 × 3.75 × 3.00 | 240 × 240 |
| 9 | The First Affiliated Hospital of Jinan University | 50 | 50 | GE Discovery MR750 3.0T | 8 channel | 2000 | 25 | 90 | 3.0 mm/1.0 mm | 35 | 200 | 3.75 × 3.75 × 4.00 | 240 × 240 |
| 10 | First Hospital of Shanxi Medical University | 50 | 33 | Siemens Tim Trio 3T | 32 channel | 2000 | 30 | 90 | 3.0 mm/1.52 mm | 32 | 212 | 3.75 × 3.75 × 4.52 | 240 × 240 |
| 11 | Department of Psychiatry, The First Affiliated Hospital of Chongqing Medical University | 32 | 29 | GE Signa 3T | 8 channel | 2000 | 30 | 90 | 5 mm | 33 | 200 | 3.75 × 3.75 × 5.00 | 240 × 240 |
| 13 | The First Affiliated Hospital of Xi’an Jiaotong University, Xi’an Central Hospital | 25 | 17 | GE Excite 1.5T | 16 channel | 2500 | 35 | 90 | 4 mm/0 mm | 36 | 150 | 4.00 × 4.00 × 4.00 | 256 × 256 |
| 14 | The Second Xiangya Hospital of Central South University | 64 | 32 | Siemens Tim Trio 3T | 32 channel | 2500 | 25 | 90 | 3.5 mm/0 mm | 39 | 200 | 3.75 × 3.75 × 3.50 | 240 × 240 |
| 15 | Department of Psychosomatics and Psychiatry, Zhongda Hospital, School of Medicine, Southeast University | 50 | 50 | Siemens Verio 3.0T MRI | 12 channel | 2000 | 25 | 90 | 4 mm/0 mm | 36 | 240 | 3.75 × 3.75 × 4.00 | 240 × 240 |
| 17 | Department of Psychiatry, The First Affiliated Hospital of Chongqing Medical University | 47 | 44 | GE Signa 3T | 8 channel | 2000 | 40 | 90 | 4.0 mm/0 mm | 33 | 240 | 3.75 × 3.75 × 4.00 | 240 × 240 |
| 19 | Anhui Medical University | 51 | 36 | GE Signa 3T | 8 channel | 2000 | 22.5 | 30 | 4.0 mm/0.6 mm | 33 | 240 | 3.44 × 3.44 × 4.60 | 220 × 220 |
| 20 | Faculty of Psychology, Southwest University | 282 | 251 | Siemens Tim Trio 3T | 12 channel | 2000 | 30 | 90 | 3.0 mm/1.0 mm | 32 | 242 | 3.44 × 3.44 × 4.00 | 220 × 220 |
| 21 | Beijing Anding Hospital, Capital Medical University | 86 | 70 | Siemens Tim Trio 3T | 32 channel | 2000 | 30 ms | 90 | 3.5 mm/0.7 mm | 33 | 240 | 3.12 × 3.12 × 4.20 | 200 × 200 |
| 22 | The Institute of Mental Health, Second Xiangya Hospital of Central South University | 30 | 20 | Philips Gyroscan Achieva 3.0T | 32 channel | 2000 | 30 | 90 | 4.0 mm/0 mm | 36 | 250 | 1.67 × 1.67 × 4.00 | 240 × 240 |
| 23 | Mental Health Center, West China Hospital, Sichuan University | 32 | 30 | Philips Achieva 3.0T TX | 8 channel | 2000 | 30 | 90 | 4.0 mm/0 mm | 38 | 240 | 3.75 × 3.75 × 4.00 | 240 × 240 |

*: The serial number of each site followed the original Yan et al. paper.

Table S2. Group differences in overall functional connectivity strength.

| Group | t | p |
| --- | --- | --- |
| MDD vs. NC | -1.814 | 0.070 |
| FEDN vs. NC | 0.302 | 0.763 |
| Recurrent vs. NC | -2.924 | 0.004 |
| FEDN vs. Recurrent | 2.495 | 0.014 |

Abbreviations: MDD: major depressive disorder; NC: normal control; FEDN: first-episode drug naïve.

Table S3. Group differences in topological metrics after regressing out overall connectivity strength.

| Contrasts | Metrics | t | p |
| --- | --- | --- | --- |
| MDD vs. NC | E_glob_ | -3.628 | < 0.001 |
|  | E_loc_ | -3.701 | < 0.001 |
|  | L_p_ | 3.231 | 0.001 |
|  | C_p_ | -2.430 | 0.015 |
| FEDN vs. NC | E_glob_ | -1.647 | 0.100 |
|  | E_loc_ | -2.107 | 0.036 |
|  | L_p_ | 2.464 | 0.014 |
|  | C_p_ | -2.087 | 0.037 |
| Recurrent vs. NC | E_glob_ | -3.317 | 0.001 |
|  | E_loc_ | -4.111 | < 0.001 |
|  | L_p_ | 3.919 | < 0.001 |
|  | C_p_ | -4.124 | < 0.001 |
| FEDN vs. Recurrent | E_glob_ | 0.806 | 0.422 |
|  | E_loc_ | 0.786 | 0.433 |
|  | L_p_ | -0.381 | 0.704 |
|  | C_p_ | 0.720 | 0.472 |

Abbreviations: MDD: major depressive disorder; NC: normal control; FEDN: first-episode drug naïve.

Table S4. Effects of medication and illness duration on topological metrics.

| Contrasts | Metrics | t | p |
| --- | --- | --- | --- |
| FED vs. FEDN | E_glob_ | -1.769 | 0.078 |
|  | E_loc_ | -1.871 | 0.063 |
|  | L_p_ | 1.823 | 0.070 |
|  | C_p_ | -2.018 | 0.045 |
| Short vs. Long Duration | E_glob_ | -1.082 | 0.280 |
|  | E_loc_ | -0.999 | 0.319 |
|  | L_p_ | 0.788 | 0.432 |
|  | C_p_ | -0.800 | 0.424 |

Abbreviations: FED: first episode on medication; FEDN: first-episode drug naïve.

Table S5. Group differences in topological metrics. Medication usage was included as an additional covariate.

| Contrasts | Metrics | t | p |
| --- | --- | --- | --- |
| MDD vs. NC | E_glob_ | -1.270 | 0.204 |
|  | E_loc_ | -1.481 | 0.139 |
|  | L_p_ | 1.734 | 0.083 |
|  | C_p_ | -1.435 | 0.152 |

Abbreviations: MDD: major depressive disorder; NC: normal control.

Table S6. Sex differences in topological metrics using the original LME model.

| Contrasts | Metrics | t | p |
| --- | --- | --- | --- |
| MDD vs. NC | E_glob_ | -3.591 | < 0.001 |
|  | E_loc_ | -3.562 | < 0.001 |
|  | L_p_ | 3.187 | < 0.001 |
|  | C_p_ | -3.303 | < 0.001 |

Abbreviations: MDD: major depressive disorder; NC: normal control.

Table S7. Group-by-sex interactions in topological metrics.

| Contrasts | Metrics | t | p |
| --- | --- | --- | --- |
| MDD vs. NC | E_glob_ | 0.385 | 0.700 |
|  | E_loc_ | 0.221 | 0.825 |
|  | L_p_ | 0.206 | 0.837 |
|  | C_p_ | -0.342 | 0.732 |

Abbreviations: MDD: major depressive disorder; NC: normal control.


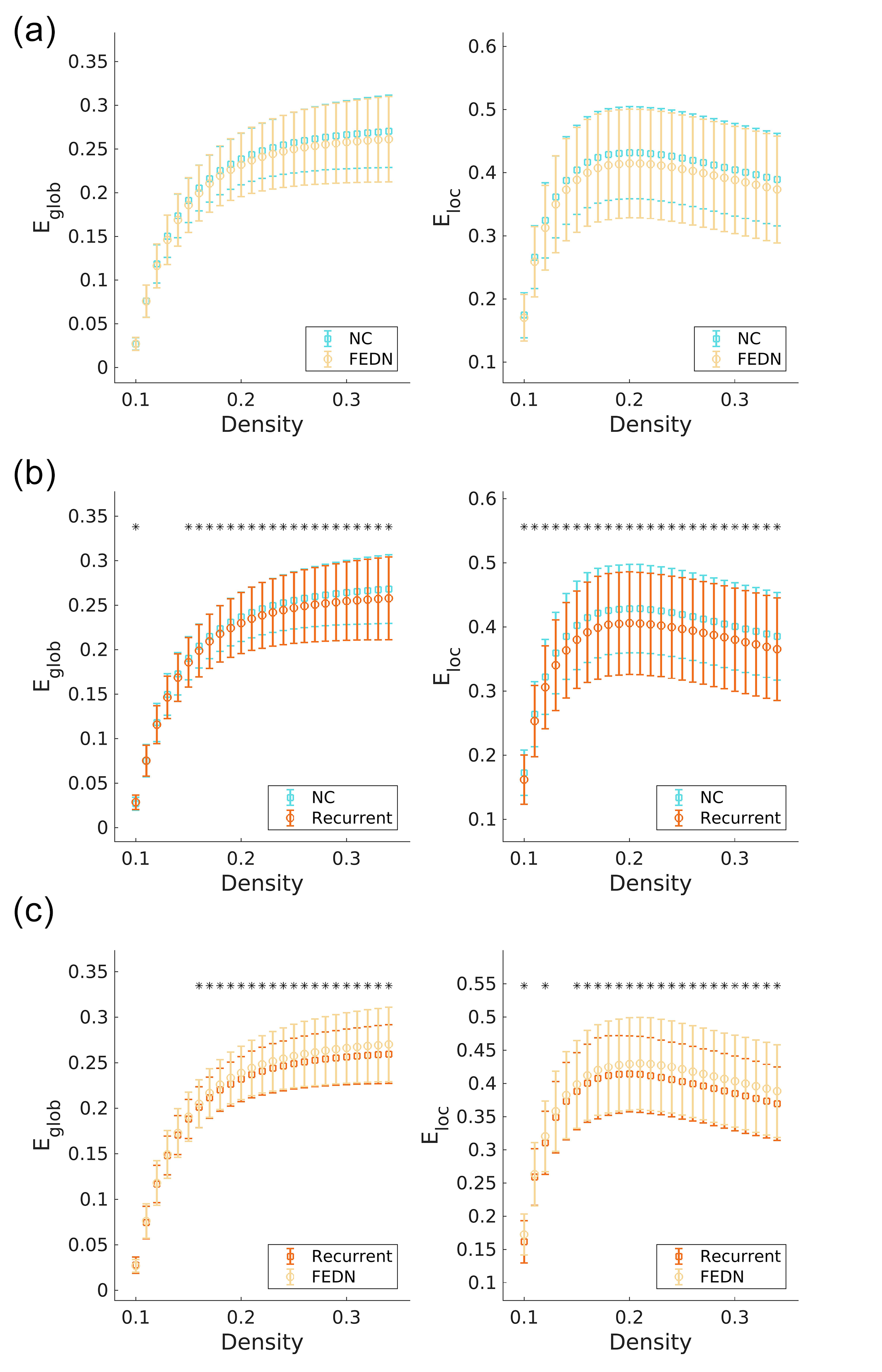


Figure S1. Subgroup effects of graph theory features (global efficiency, E_glob_, and local efficiency, E_loc_) across all density levels. Each point and error bar denote the mean and standard deviation at each density level, respectively. Asterisks indicate a significant difference at this density threshold. (a), first-episode drug naïve (FEDN) major depressive disorder (MDD) patients vs. normal controls (NC). (b), recurrent patients with MDD vs. NCs. (c), FEDN patients vs. recurrent patients with MDD.


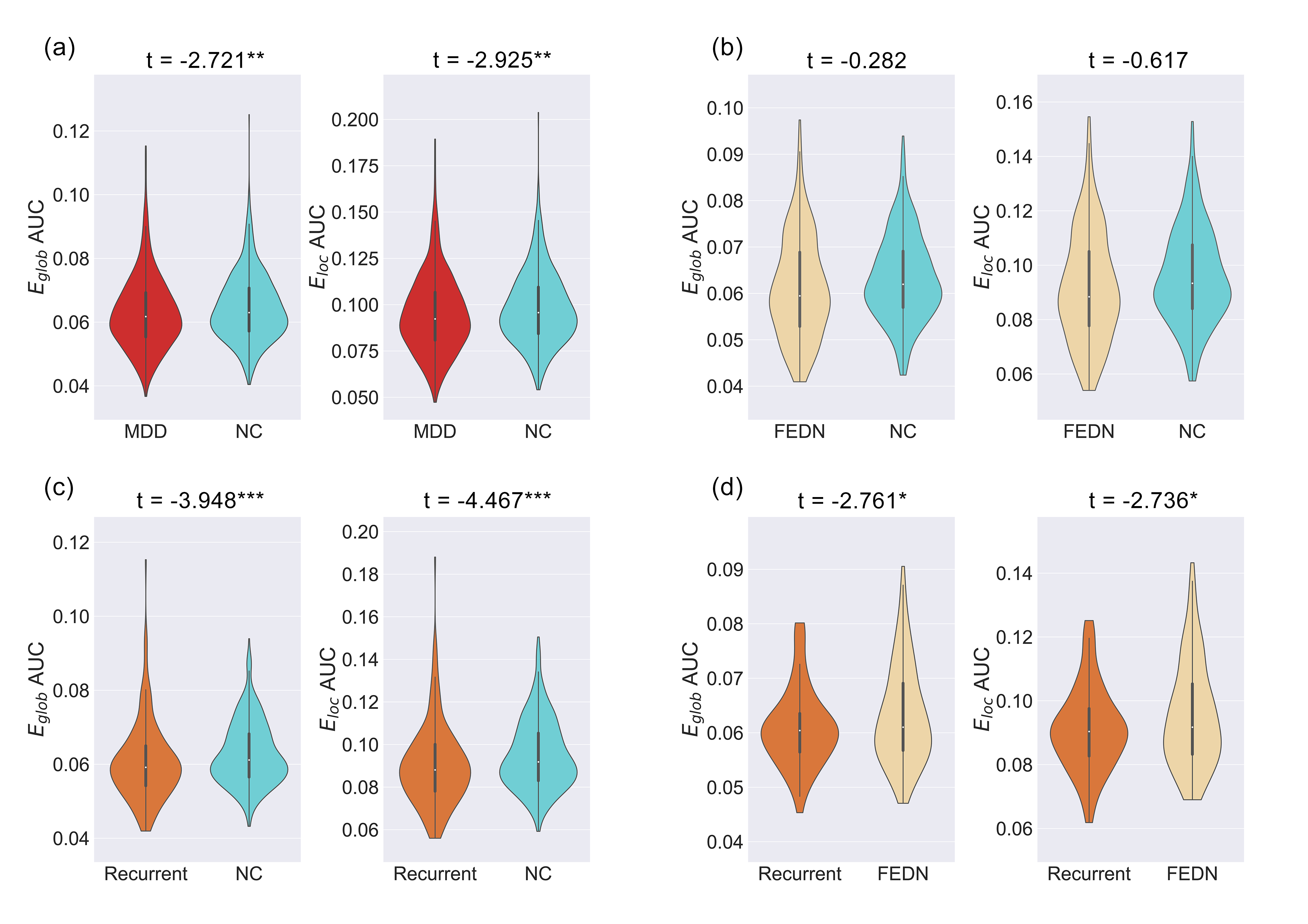


Figure S2. Violin plots depicting the results of global and local efficiency (E_glob_ and E_loc_, respectively) from the “scrubbing” pipeline. Distributions of areas under curve (AUCs) are depicted. (a) Major depressive disorder (MDD) patients vs. normal controls (NCs). (b) First-episode drug naïve (FEDN) major depressive disorder (MDD) patients vs. normal controls (NCs). (c), recurrent patients with MDD vs. NCs. (d), Recurrent patients with MDD vs. FEDN patients. *: p < 0.05, **: p < 0.01, ***: p < 0.001.


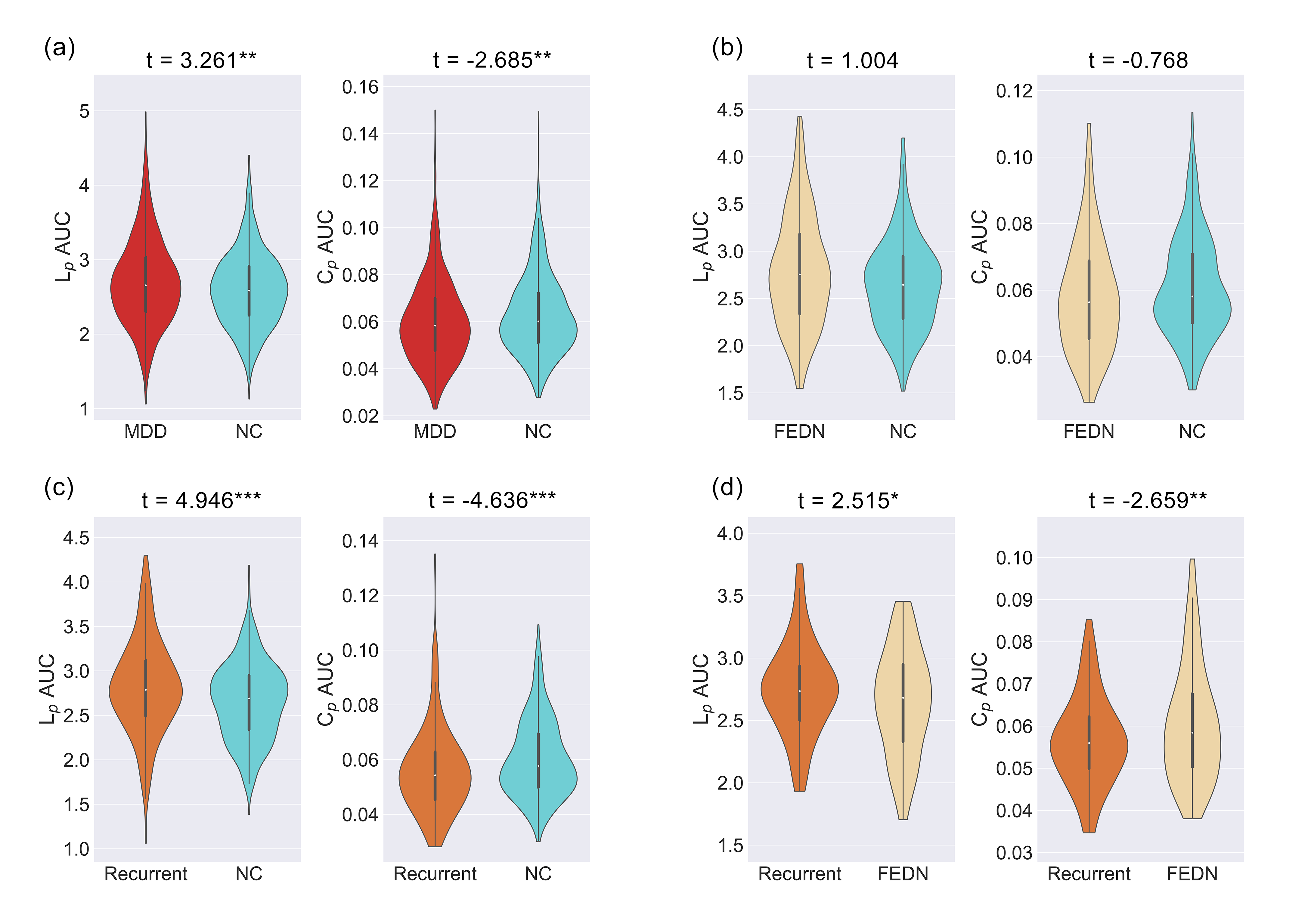


Figure S3. Violin plots depicting the results of the characteristic path length (L_p_) and clustering coefficient (C_p_) from the “scrubbing” pipeline. Distributions of areas under curve (AUCs) are depicted. (a) Major depressive disorder (MDD) patients vs. normal controls (NCs). (b) First-episode drug naïve (FEDN) major depressive disorder (MDD) patients vs. normal controls (NCs). (c), Recurrent patients with MDD vs. NCs. (d), Recurrent patients with MDD vs. FEDN patients. *: p < 0.05, **: p < 0.01, ***: p < 0.001.


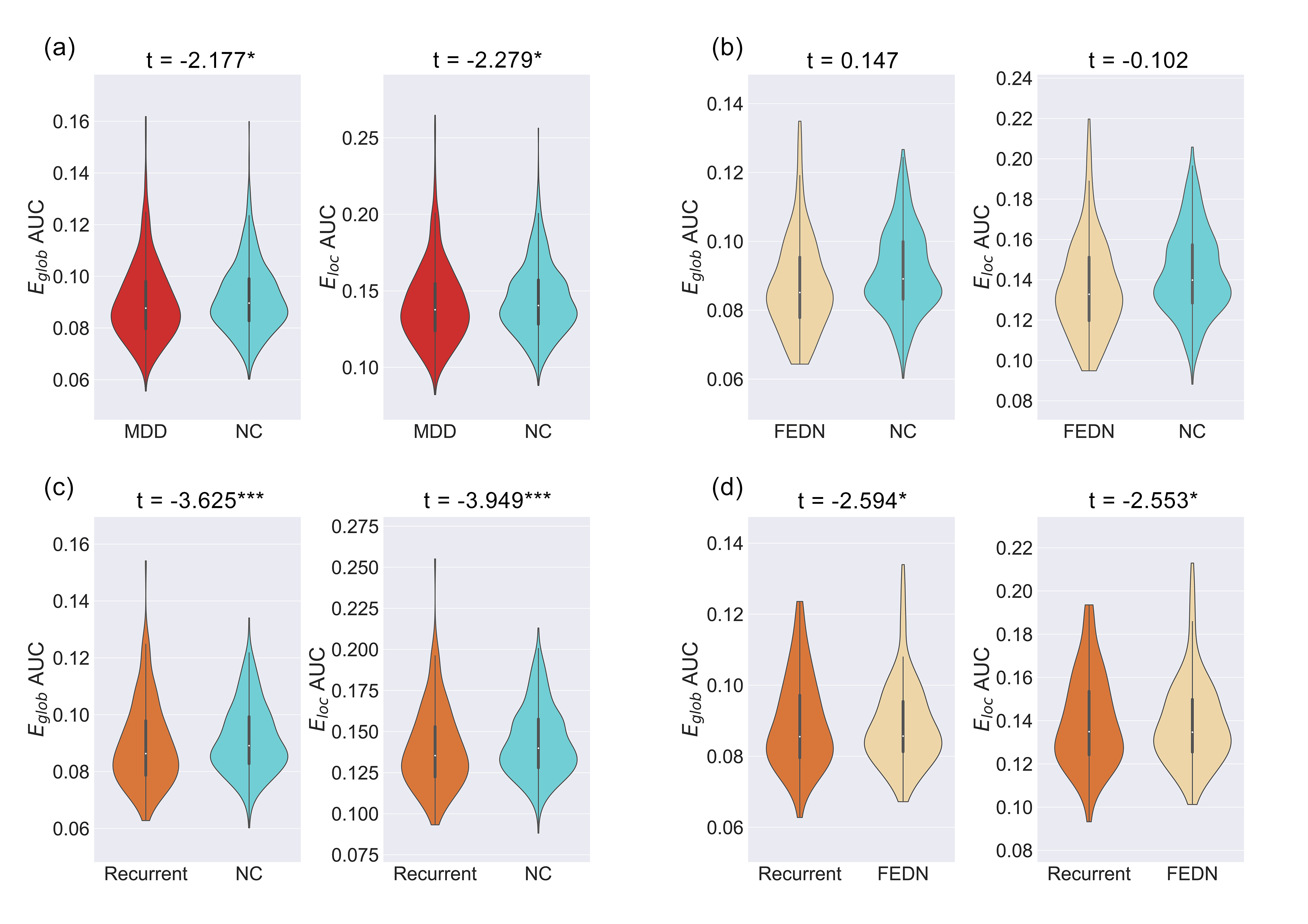


Figure S4. Violin figures depicting the results of global and local efficiency (E_glob_ and E_loc_, respectively) calculated with Craddock’s 200 functional clustering atlas. Distributions of areas under curve (AUCs) are depicted. (a) Major depressive disorder (MDD) patients vs. normal controls (NCs). (b) First-episode drug naïve (FEDN) major depressive disorder (MDD) patients vs. normal controls (NCs). (c), Recurrent patients with MDD vs. NCs. (d), Recurrent patients with MDD vs. FEDN patients. *: p < 0.05, **: p < 0.01, ***: p < 0.001.


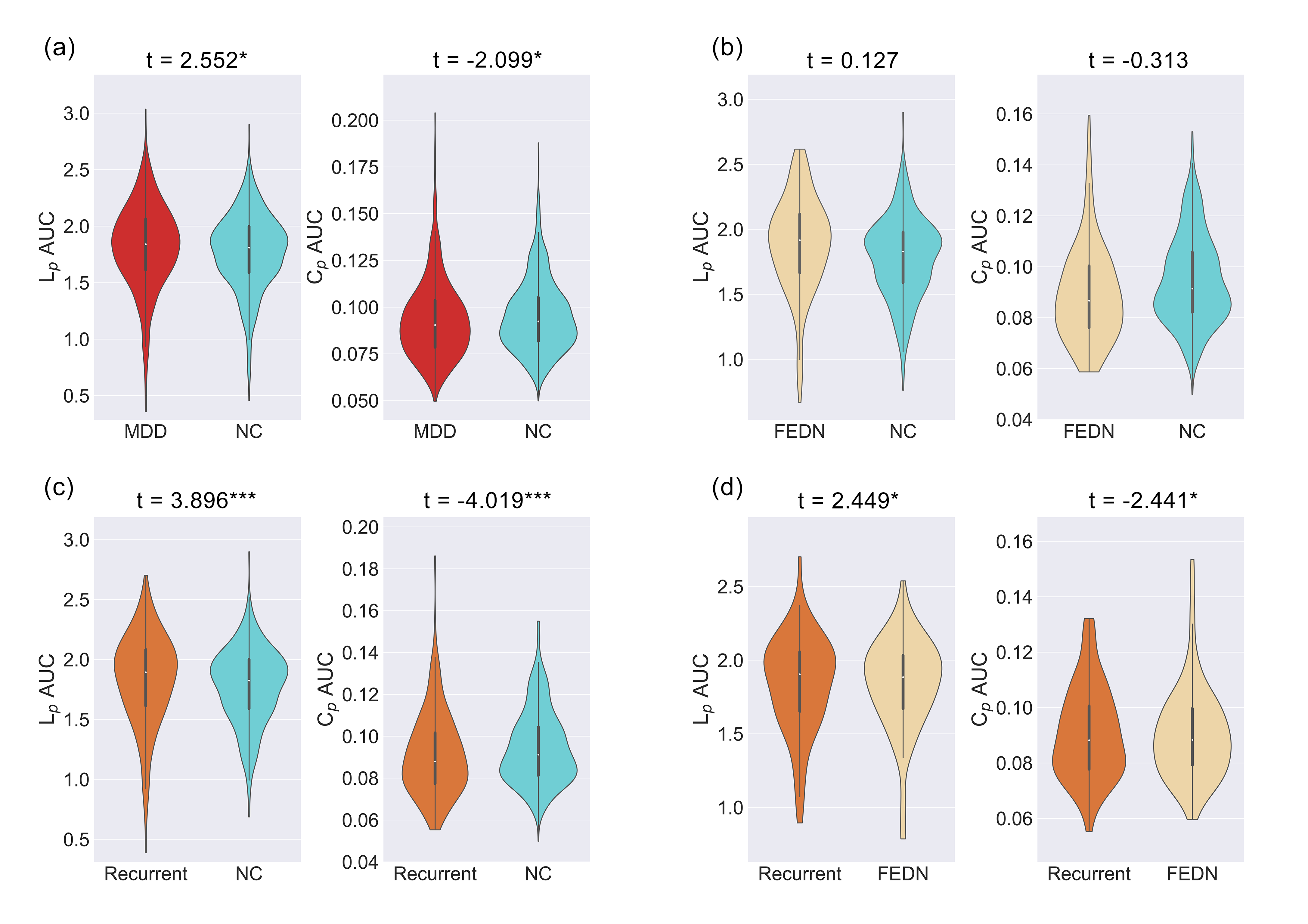


Figure S5. Violin plots depicting results of characteristic path length (L_p_) and clustering coefficient (C_p_) calculated with the Craddock’s 200 functional clustering atlas. Distributions of areas under curve (AUC) are depicted. (a) Major depressive disorder (MDD) patients vs. normal controls (NC). (b) First episode drug naïve (FEDN) major depressive disorder (MDD) patients vs. normal controls (NC). (c), recurrent patients with MDD vs. NCs. (d), Recurrent patients with MDD vs. FEDN patients. *: p < 0.05, **: p < 0.01, ***: p < 0.001.
